# Supplementary material for: Perceived Time Shapes Physical Fatigue Accumulation and Its Neural Oscillatory Correlates
Source: Adv Sci (Weinh). 2026 Sep 7:e77165. Online ahead of print. doi: 10.1002/advs.77165 (PMC13548731; doi:10.1002/advs.77165)
Supplement: Supplementary file 1 [file ADVS-9999-e77165-s001.docx]

## Perceived time shapes physical fatigue accumulation and its neural oscillatory correlates

Pierre-Marie Matta, Julien Duclay, Robin Baurès, Andrea Alamia

## Supplementary Information

## Supplementary Results

Our results show that perceived time can significantly influence the accumulation of physical fatigue and that the frontal oscillatory dynamics are robustly modulated by perceived rather than actual time. Starting from the assumption that PFC is involved in belief representation (Geuter et al., 2017), we hypothesized that participants with the strongest deceptive effect in the frontal electrodes would also exhibit the largest EMG effect. To test our hypothesis, we split the participants into two groups via a median split computed on the normalized θ-power difference between the two sessions with the same perceived time. The first split separated participants based on the normalized θ-power difference computed between sessions that shared a perceived short duration (P10) but differed in real duration. In a second median split, the same variable was computed for sessions that shared a perceived long duration (P12) but differed in real duration. These power differences were normalized by the difference between the P12R12 and P10R10 conditions to account for the power difference observed under normal clock conditions. In both cases, smaller differences correspond to a larger deceptive effect, as the θ power modulation in frontal electrodes follows the perceived rather than the actual time (‘Subjective-time’ (ST) group). Conversely, participants with larger power differences would rely more on actual than perceived time (‘Objective-time’ (OT) group). Note that we used the θ power difference to perform our median split because of its well-established relationship with cognitive control (Cavanagh & Frank, 2014).

Figure S1 – Physical fatigue accumulation in the ‘Subjective-time’ subgroup

A. EMG modulations over the main task. EMG is expressed in normalized RMS over contractions, whose increase reflects physical fatigue accumulation. Panels contain the ‘Subjective-time’ participants only (N = 12) (based on the deception effects measured in the θ-band, see text for details). Panel on the left was computed on frontal θ absolute sum power difference between sessions sharing the same perceived short duration (P10) and panel on the right between sessions sharing the same perceived long duration (P12). For both panels, the lines represent the averaged distribution, and the error bars are the standard error of the mean. B. EMG differences. EMG differences were computed for each contrast. The left panel illustrates perceived-time effects by holding real duration constant and contrasting long versus short perceived durations, whereas the right panel illustrates real-time effects by holding perceived duration constant and contrasting long versus short real durations. All panels represent differences in the slope of the EMG-RMS across bins. For all panels, the bar plots represent model-based estimates of condition differences, with error bars indicating 95% confidence intervals. Intervals not crossing zero indicate statistically significant effects at a Bonferroni-corrected alpha of 0.05.

For all subgroups, a linear model with a simple contraction effect (linear) was sufficient, as adding a second-degree spline did not improve model fit (all p > .15). In the ST subgroup defined on the θ P10 difference, significant main effects of contraction number (F_(1, 11)_ = 15.91, p = .0021), perceived time (F_(1, 4767)_ = 6.18, p = .013), and real time (F_(1, 4767)_ = 35.20, p < .0001) were observed. Significant interactions were found between contraction number and perceived time (F_(1, 4758)_ = 88.76, p < .0001), between contraction number and real time (F_(1, 4748)_ = 12.42, p = .0004), between perceived and real time (F_(1, 4764)_ = 17.11, p < .0001), and a three-way interaction among contraction number, perceived time, and real time (F_(1, 4758)_ = 15.45, p < .0001). Post-hoc contrasts on the estimated marginal slopes indicated that EMG increased more for long perceived time, both when real time was short (p < .001, d_z_ = 1.12) and long (p < .0001, d_z_ = 2.73), indicating that EMG can be both slowed down and accelerated, according to the clock calibration. Accordingly, there was no significant influence of real duration when perceived time was short (p = 1, d_z_ = 0.08, BF = 0.29). Finally, when perceived time was long, EMG rate was still modulated by real time (p <.0001, d_z_ = 1.52) (Fig. 5A, 5B).

In the ST subgroup defined on the θ P12 difference, contraction number (F_(1, 11)_ = 25.13, p < .0001) and real time (F_(1, 4767)_ =38.71, p < .0001) showed significant main effects. Interactions between contraction number and perceived time (F_(1, 4758)_ = 63.50, p < .0001), between contraction number and real time (F_(1, 4758)_ = 57.39, p < .0001), between perceived and real time (F_(1, 4765)_ = 6.83, p = .009), and the three-way interaction (F_(1, 4758)_ = 16.97, p < .0001) were also significant. EMG increased more at long perceived time both when real time was short (p = 0.026, d_z_ = 0.79) and long (p < .0001, d_z_ = 2.47), supporting a symmetric EMG modulation by perceived time. Finally, real duration provided inconclusive evidence when perceived time was short (p = 0.058, d_z_ = 0.71, BF = 0.46) and had a significant influence under long perceived time (p < .0001, d_z_ = 2.39) (Fig. 5A, 5B).

In the OT subgroup defined on the θ P10 difference, significant main effects of contraction number (F_(1, 11)_ = 33.09, p < .0001) and perceived time (F_(1, 4764)_ = 68.78, p < .0001) were observed, along with significant interactions between contraction number and perceived time (F_(1, 4758)_ = 4.23, p = .040), between contraction number and real time (F_(1, 4758)_ = 50.32, p < .0001), and between perceived and real time (F_(1, 4766)_ = 146.49, p < .0001). Post-hoc contrasts on the estimated marginal means showed that EMG was greater with long perceived time when real time was short (p < .0001, d_z_ = 6.29) but lower with long perceived time when real time was long (p < .0001, d_z_ = 1.47), suggesting an opposite pattern to the ST group. Similarly, EMG was greater with long real time when perceived time was short (p < .0001, d_z_ = 6.17) but lower with long real time when perceived time was long (p < .0001, d_z_ = 1.33).

Finally, in the OT subgroup defined on the θ P12 difference, significant main effects of contraction number (F(1, 11) = 36.57, p < .0001) and perceived time (F(1, 4764.3) = 66.89, p < .0001) were observed, along with a significant interaction between contraction number and real time (F(1, 4757.9) = 8.13, p = .004) and a significant interaction between perceived and real time (F(1, 4765.1) = 196.54, p < .0001). Post-hoc contrasts on the estimated marginal means showed that EMG was greater with long perceived time when real time was short (p < .0001, d_z_ = 7.19) but lower with long perceived time when real time was long (p < .0001, d_z_ = 2.04), again in the opposite direction to the ST group. Similarly, EMG was greater with long real time when perceived time was short (p < .0001, d_z_ = 5.44) but lower with long real time when perceived time was long (p < .0001, d_z_ = 3.43).

While this analysis remains exploratory and is partially limited by a median split, a small sample size (n = 12 per group), and the risk of circular inference reasoning, these results might suggest that the frontal power changes play a key role in the effectiveness of the deceptive effect on fatigue accumulation, as measured by EMG. Interestingly, it reveals that, in the ST group, fatigue can be slowed down or accelerated depending on the clock calibration.

Figure S2 – Topographic maps theta/beta

Median scalp topographies of theta and beta relative power (normalized to the first contractions) averaged over 25-contraction bins (columns) across the four experimental conditions (rows: P10R10, P10R12, P12R12, P12R10). These maps allow visual inspection of the spatial distribution of the effects reported in the main text.

Table S1 – Session order counterbalancing table

| **Participant** | **Session 1** | **Session 2** | **Session 3** | **Session 4** |
| --- | --- | --- | --- | --- |
| 1 | P10R10 | P12R12 | P12R10 | P10R12 |
| 2 | P12R12 | P10R12 | P12R10 | P10R10 |
| 3 | P12R10 | P12R12 | P10R12 | P10R10 |
| 4 | P10R12 | P10R10 | P12R12 | P12R10 |
| 5 | P10R12 | P12R10 | P10R10 | P12R12 |
| 6 | P10R10 | P10R12 | P12R12 | P12R10 |
| 7 | P10R10 | P12R12 | P12R10 | P10R12 |
| 8 | P12R12 | P12R10 | P10R12 | P10R10 |
| 9 | P12R12 |  |  |  |
| 10 | P12R10 | P10R12 | P12R12 | P10R10 |
| 11 | P12R12 | P10R10 | P10R12 | P12R10 |
| 12 | P10R12 | P12R12 | P10R10 | P12R10 |
| 13 | P12R10 |  |  |  |
| 14 | P10R10 | P12R10 | P10R12 | P12R12 |
| 15 | P10R12 | P10R10 | P12R10 | P12R12 |
| 16 | P12R10 | P12R12 | P10R10 | P10R12 |
| 17 | P10R10 | P10R12 | P12R10 | P12R12 |
| 18 | P12R12 | P12R10 | P10R12 | P10R10 |
| 19 | P12R10 | P10R10 | P12R12 | P10R12 |
| 20 | P12R12 | P10R12 | P10R10 | P12R10 |
| 21 | P12R10 | P10R12 | P10R10 | P12R12 |
| 22 | P10R12 |  |  |  |
| 23 | P10R12 | P12R10 | P12R12 | P10R10 |
| 24 | P10R10 | P12R10 | P12R12 | P10R12 |
| 25 | P10R12 | P12R12 | P12R10 | P10R10 |
| 26 | P12R12 |  |  |  |
| 27 | P12R12 | P10R10 | P12R10 | P10R12 |
| 28 | P12R10 | P10R10 | P10R12 | P12R12 |

Note. This table presents the order in which the four experimental sessions (P10R10, P10R12, P12R12, P12R10) were administered to each of the 28 participants recruited. All 24 possible orderings of the four sessions were initially assigned across the 28 recruited participants in a fully counterbalanced design. Following the exclusion of 4 participants, the final sample covers 22 of the 24 possible orders, with two orders each represented twice, and two orders not represented.

Table S2 - Data transparency matrix

| **Component /** *Variable* | **Current paper** | **Matta et al., 2025** |
| --- | --- | --- |
| **Participants** | **Same cohort** | **Same cohort** |
| **Sessions** | **4 sessions** | **2 sessions** |
| **Time Deception** | **Yes** | **No** |
| **Main research question** | **Time Deception effect** | **Beta oscillations in fatigue** |
| **Main statistical approach** | **LMMs over contractions** | **ANOVAs on binned data** |
| **Reported results** | **Non-overlapping** | **Non-overlapping** |
| *Maximal Voluntary Torque* | *Yes* | *Yes* |
| *EMG* | *Yes* | *Yes* |
| *Individualized β-band Cz* | *Yes* | *Yes* |
| *Individualized β-band frontal* | *Yes* | *Yes* |
| *Resting State EEG β-band Cz* | *Yes* | *Yes* |
| *Cz-EMG Correlation* | *Yes* | *Yes* |
| *θ-band frontal* | *Yes* | *No* |
| *Cz-Frontal Correlation* | *Yes* | *No* |
| *θ-band Frontal based median split* | *Yes* | *No* |
| *Interoceptive awareness correlation* | *Yes* | *No* |

Note. This table details the overlap between the dataset used in the present study and that reported in Matta et al. (2025). Two of the four experimental sessions (P10R10 and P12R12, the two normal clock conditions) were previously analyzed in Matta et al. (2025) in the context of a distinct research question focusing on beta-band oscillatory dynamics during physical fatigue. The data from the two sessions involving biased clock conditions (P10R12 and P12R10) have not been reported elsewhere. The two studies used different contrasts and modeling strategies; in particular, the hypotheses, results, and conclusions of the two manuscripts are distinct.

Table S3 – BIC models comparison tables for longitudinal variables

| **Variable** | **M1 df=1** | **M1 df=2** | **M2 df=1** | **M2 df=2** | **M3 df=1** | **M3 df=2** | **M4 df=1** | **M4 df=2** |
| --- | --- | --- | --- | --- | --- | --- | --- | --- |
| EMG | 52133.3 | 52162.5 | 51373.9 | 51402.5 | 47119.6 | 47144.1 | 47124.0 | 47149.0 |
| θ Front. | 71339.4 | 71371.0 | 71328.3 | 71360.0 | 70782.8 | 70814.2 | 70791.0 | 70823.0 |
| Iβ Front | 82928.7 | 82758.1 | 82937.9 | 82767.3 | 81375.5 | 81164.0 | 81385.0 | 81173.0 |
| Iβ Cz | 75009.7 | 75035.0 | 75000.5 | 75026.0 | 74165.5 | 74189.9 | 74174.0 | 74198.0 |

Note. This table report the BIC values used to select the optimal model structure and contraction term degree for each dependent variable. It presents the comparison of four random effects structures (M1–M4) across both linear (df=1) and spline (df=2) contraction terms for variables measured longitudinally across contractions. The four structures are defined as follows: M1 (baseline): DV ~ ns(NumContraction_z, df=d) × Perceived × Real + (1 + NumContraction_z || Participant); M2 (session order as fixed effect): DV ~ ns(NumContraction_z, df=d) × Perceived × Real + Session_Order + (1 + NumContraction_z || Participant); M3 (session order as random slope): DV ~ ns(NumContraction_z, df=d) × Perceived × Real + (1 + NumContraction_z + Session_Order || Participant); M4 (session order as fixed effect and random slope): DV ~ ns(NumContraction_z, df=d) × Perceived × Real + Session_Order + (1 + NumContraction_z + Session_Order || Participant). The model with the lowest BIC (highlighted in blue) was retained as the primary model.

Table S4 – BIC models comparison tables for pre-post variables

| **Variable**  **Pre-Post** | **M1** | **M2** | **M3** | **M4** |
| --- | --- | --- | --- | --- |
| MVT | 682.8 | 686.5 | 691.5 | 695.3 |
| Iβ Cz | 805.7 | 807.8 | 807.3 | 810.3 |

Note. This table presents the equivalent comparison than for Table S3, but for variables measured as pre-post differences, for which no contraction term is included. The model with the lowest BIC (highlighted in blue) was retained as the primary model.
